# Distinct and Competitive Regulatory Patterns of Tumor Suppressor Genes and Oncogenes in Ovarian Cancer

**DOI:** 10.1371/journal.pone.0044175

**Published:** 2012-08-30

**Authors:** Min Zhao, Jingchun Sun, Zhongming Zhao

**Affiliations:** 1 Department of Biomedical Informatics, Vanderbilt University School of Medicine, Nashville, Tennessee, United States of America; 2 Department of Cancer Biology, Vanderbilt University School of Medicine, Nashville, Tennessee, United States of America; 3 Department of Psychiatry, Vanderbilt University School of Medicine, Nashville, Tennessee, United States of America; 4 Center for Quantitative Sciences, Vanderbilt University, Nashville, Tennessee, United States of America; Niels Bohr Institute, Denmark

## Abstract

**Background:**

So far, investigators have found numerous tumor suppressor genes (TSGs) and oncogenes (OCGs) that control cell proliferation and apoptosis during cancer development. Furthermore, TSGs and OCGs may act as modulators of transcription factors (TFs) to influence gene regulation. A comprehensive investigation of TSGs, OCGs, TFs, and their joint target genes at the network level may provide a deeper understanding of the post-translational modulation of TSGs and OCGs to TF gene regulation.

**Methodology/Principal Findings:**

In this study, we developed a novel computational framework for identifying target genes of TSGs and OCGs using TFs as bridges through the integration of protein-protein interactions and gene expression data. We applied this pipeline to ovarian cancer and constructed a three-layer regulatory network. In the network, the top layer was comprised of modulators (TSGs and OCGs), the middle layer included TFs, and the bottom layer contained target genes. Based on regulatory relationships in the network, we compiled TSG and OCG profiles and performed clustering analyses. Interestingly, we found TSGs and OCGs formed two distinct branches. The genes in the TSG branch were significantly enriched in DNA damage and repair, regulating macromolecule metabolism, cell cycle and apoptosis, while the genes in the OCG branch were significantly enriched in the ErbB signaling pathway. Remarkably, their specific targets showed a reversed functional enrichment in terms of apoptosis and the ErbB signaling pathway: the target genes regulated by OCGs only were enriched in anti-apoptosis and the target genes regulated by TSGs only were enriched in the ErbB signaling pathway.

**Conclusions/Significance:**

This study provides the first comprehensive investigation of the interplay of TSGs and OCGs in a regulatory network modulated by TFs. Our application in ovarian cancer revealed distinct regulatory patterns of TSGs and OCGs, suggesting a competitive regulatory mechanism acting upon apoptosis and the ErbB signaling pathway through their specific target genes.

## Introduction

Cancer is characterized by uncontrolled cell growth, which is caused by the accumulated genomic mutations in genes that normally play important roles in controlling cell proliferation and apoptosis [1]. Two major groups of protein-coding genes influence cancer cell growth in opposite ways. The first group of genes encode tumor suppressors, whose loss of function contributes to the development of cancer [2]. The second group of genes are oncogenes, whose gain of function can trigger cancer development [3]. Hereafter, we abbreviated these two types of genes as TSGs and OCGs. Many TSGs are the “guardian of the cell” because of their critical roles in cell cycle checkpoints and inducing apoptosis [2], [4]. For instance, the well-known TSGs *RB* and *TP53* are regarded as anti-oncogenes because of their effects on actions against known oncogenes in cell growth [5]. In a normal cell, OCGs are located on chromosomes as proto-oncogenes. When activated by point mutations or other mechanisms like gene amplification, proto-oncogenes may be converted into OCGs to stimulate cell proliferation and promote cell survival by interfering with apoptosis [3].

In the past few decades, a substantial number of TSGs and OCGs were characterized according to their functions in cell proliferation and apoptosis [2], [3], [4]. However, the underlying molecular mechanisms for these TSGs and OCGs to regulate biological processes at transcription level are still not clear, especially at the systems and cellular levels. It is well-known that DNA-binding transcription factors (TFs) play major roles in a gene’s transcriptional regulation [6]. TF activities are mainly regulated by other molecules at the post-translational level [7]. Previously, studies have shown that TSGs, such as *RB1* and *KL*, can affect the activity of TFs or growth factors as their post-translational modulators [8], [9]. In cancer tumorigenesis, the majority of TSGs and OCGs do not belong to the category of TFs; thus, they cannot directly regulate gene expression. Therefore, modulating TFs at the post-translational level may provide a mechanism for TSGs and OCGs to regulate gene expression indirectly.

Ovarian cancer (abbreviated as OVC in this study) is the fifth leading cause of cancer-related mortality with a prevalence of 1.4% to 2.5% in U.S. women [10]. The search for convincing candidate genes in the past decade, although far from complete or conclusive, has provided the foundation for systematic analyses of their genetic contributions to OVC [11]. Additionally, genome-scale technologies have generated vast quantities of gene expression profiling and other genetic and genomic data from hundreds of OVC samples [12], [13], [14], [15]. These genetic and high-throughput genomic data have provided us an opportunity to identify a critical regulatory network that is vital to cancer development [16], [17]. Moreover, the carcinogenesis of the ovary involves many specific etiological factors such as hormones and ovulation [18], which brings into question how the gene regulatory network integrates signals to respond to hormone stimuli. Our hypothesis here was that a systematic integration of TFs and their potential modulators (TSGs and OCGs) provides an efficient approach to discover a gene regulatory network in OVC. This regulatory network may provide the novel regulation model of TSGs, OCGs and TFs on gene expression in critical tumorigenesis processes such as cell cycle and hormone stimulus.

Here, we present a computational approach to construct a hierarchical regulatory network from protein-protein interactions (PPIs) and gene expression data using TFs as bridges to link important modulators (TSGs and OCGs) to their potential target genes. We applied this approach to construct a three-layer regulatory network in OVC, in which the top layer included 29 TSGs and 13 OCGs, the middle layer included 15 TFs, and the bottom layer included 65 joint target genes. Further regulatory profile clustering analyses divided TSGs and OCGs into two distinct branches. The TSGs were mainly involved in DNA damage and repair, cell cycle, and apoptosis, while OCGs were mainly clustered together in ErbB signaling transduction and response to hormone stimuli. Additionally, OCG-specific target genes were enriched in negative apoptosis regulators, while TSG-specific target genes were enriched in ErbB signaling pathways. These results revealed a distinct function pattern of TSGs and OCGs, not only by their own opposite functions in cancer development but also by the opposite enriched functions of their specific target genes. We have, for the first time, reported a competitive regulation pattern of TSGs and their targets investigated in comparison to OCGs and their targets; in this reported finding, we found the TSGs, OCGS, along with their respective targets, have the tendency to react in opposition upon apoptosis and the ErbB signaling pathway. Further investigation of this finding is warranted.

## Materials and Methods

### Gene Collection and Curation of TSGs, OCGs and TFs in OVC

To comprehensively collect the OVC-related genes, we parsed and curated fourteen data sources, including the cancer mutation database Catalogue of Somatic Mutations in Cancer (COSMIC, version 55) [19], Online Mendelian Inheritance in Man (OMIM, October, 2011) [20], Genetic Association Database (GAD, October, 2011) [21], the database of Functional Census of Human Cancer Gene (F-CENSUS, October, 2011) [22], the online Dragon Database for Exploration of Ovarian Cancer Genes (DDOC, October, 2011) [23], one comprehensive expert review on OVC-related genes from Nature Reviews Cancer [11], the literature database Generif [24], published genome-wide association studies [25], [26], [27], and six candidate gene lists produced by large-scale genomic platforms on OVC from The Cancer Genome Atlas (TCGA) [12]. The details for gene collection on each data source are described in Text S1, and some approaches of gene collection and annotations were also successfully applied in other diseases for candidate gene prioritization [28], [29], [30]. Finally, 1257 non-redundant OVC related genes were curated for follow-up analysis (Table S1).

We manually curated TSGs and OCGs from classical reviews on OVC and general cancer [2], [4], [11], [31] and extracted known human TFs from the TRANSFAC professional database (Release 2011.4) [32]. Among 1257 OVC genes, 100 unique regulators were assigned, including 35 TSGs, 15 OCGs, and 50 TF genes.

### Network Topological Analyses and Extraction a Subnetwork Centered by TSGs, OCGs and TFs from Human Interactome

We downloaded undirected human protein-protein interaction (PPI) data from the Protein Interaction Network Analysis (PINA) platform (June, 2011) [33]. In PINA, the data included self-interactions, predicted interactions by computational methods, and interactions between human proteins and proteins from other species. In our pipeline, we only utilized the non-redundant human PPIs with experimental supports after we removed predicted PPI and self-interactions, as well as PPIs involving proteins from other species. This process generated a human PPI network with 11,654 nodes (proteins) and 72,630 links (PPIs).

To construct a subnetwork centered by TSGs, OCGs and TFs and have an overview for the topological network properties of these OVC genes, we first mapped all the related genes to the human PPI network. For comparison, we compiled five gene lists to perform network topological analyses. The first dataset included 467 known cancer genes from the Sanger Cancer Gene Census list [34], among which 378 genes were mapped to the human PPI network. Next, we divided our collected 1257 OVC genes into four groups: known TSGs, known OCGs, TFs and the remaining common OVC genes. In total, 33 TSGs, 14 OCGs, 50 TF genes, and 905 common OVC genes were mapped to the PPI network. Next, we calculated three basic topological measures for the five gene lists. These measures included degree, betweenness centrality, and closeness centrality using the software Cytoscape [35]. The degree measures the connections of each protein in the human PPI network [36]. The betweenness centrality represents how frequently a protein locates on all shortest paths between two other proteins [36]. Closeness centrality, also called shortest-path distance, indicates the shortest steps for one node to reach another [36]. To compare these topological properties among the five gene lists, we performed two-tailed Kolmogorov-Smirnov tests (KS tests) implemented in the R package 2.13.2 [37].

To evaluate the significance of network properties of each OVC TSG, OCG and TF in the human PPI network, we applied an empirical re-sampling approach. Here, we take the TSG gene list as an example. First, for the 33 TSGs mapped to the human PPI network, we randomly selected 33 nodes from any of the 1257 OVC genes in the human PPI network and calculated the three topological properties (degree, betweenness and closeness). We repeated this randomization process 10,000 times. Next, we counted the number of random selected node sets (N) whose average degree, betweenness or closeness were higher than the observed average degree, betweenness and closeness, respectively. Lastly, we calculated their empirical *P-*value using the N/10000 for the three types of topological properties, respectively. We applied similar approaches to 14 OCGs, 50 TFs and 97 regulatory genes (33 TSGs, 14 OCGs and 50 TF genes). The summarized *P-*values were listed in Table S6.

Aside from these topological analyses, we extracted 2024 direct interactors of the 97 regulatory genes (33 TSGs, 14 OCGs and 50 TF genes) from the human PPI network to form a subnetwork comprising of 2121 genes for further analysis.

### Construction of a Hierarchical Regulatory Network Based on Gene Expression Profiles from TCGA

Recently, TCGA investigators examined gene expression from 489 high-grade serous OVC samples using three gene expression microarray platforms (Affymetrix Exon 1.0 array, Agilent 244 K whole genome expression array, and Affymetrix HT-HG-U133A array) [12]. Next, they normalized and estimated the expression for each sample and gene on each platform separately. After subtracting the mean value across samples for the same gene, they divided the expression value by the standard deviation across samples and obtained relative gene expression scores. Lastly, the relative expression data from three platforms were integrated into a single, unified data set of 11,864 genes using a factor analysis model without batch effects [12], [38]. The final gene expression data downloaded from the TCGA website is formatted as a matrix, which is one row for each gene and one column for each of the samples (https://tcga-data.nci.nih.gov/docs/publications/ov_2011/).

Among the 2121 genes in the subnetwork centered by OVC TSGs, OVC OCGs and OVC TFs, 352 genes (29 TSGs, 13 OCGs, 36 TF genes, and 274 interacting genes) overlapped the 11,864 gene expression profiles from TCGA. Next, we utilized the software MINDy (Modulator Inference by Network Dynamics) to predict the regulatory relationship between TSGs, OCGs and TFs. MINDy was used to identify modulators of TFs with expression profiles at the post-translational level based on conditional mutual information [39]. MINDy requires four inputs, including a gene expression matrix, a TF of interest, a list of potential modulator genes, and a list of potential TF targets. Therefore, an expression matrix with 352 genes in each row and 489 OVC samples in each column was collected as the first input for the software MINDy. The TFs of interest were the 36 TF genes from our extracted subnetwork. The potential modulators contained 29 TSGs and 13 OCGs. The remaining 274 interacting genes in our subnetwork were regarded as potential TF targets.

To reduce the false positives of TF-target relationships inferred by MINDy, we further predicted TF-target pairs using MATCH™ with a core score of 1.00 and a matrix score of 0.95 [32], [40]. Take the example of TF HMGA2: MINDy predicted 29 TSGs and 13 OCGs to regulate 106 and 79 target genes via modulating HMGA2, respectively. We further predicted 82 target genes from the 1257 OVC-related genes using MATCH™. After comparing the 82 predicted target genes for HMGA2, only 18 and 13 unique genes were co-regulated by the 29 TSGs and 13 OCGs, respectively. Finally, the overlapping TF-target regulations formed the output network containing 29 TSGs, 13 OCGs, 15 TF genes and 65 joint target genes. In the regulatory network, 3 TSGs, 3 OCGs, and 4 TF genes were also inferred as target genes for other TFs. Therefore, 112 unique genes were integrated into the network. In addition, the 4 known TF genes *FOXM1*, *MSX1*, *PPARG* and *STAT5A*, were assigned as target genes in our network, as they did not regulate any genes in this regulatory network. The final network visualization of 112 genes was performed using the Cytoscape software [35].

### Construction and Clustering of TF and Target Gene Profiles of TSGs and OCGs

To analyze the downstream target genes of TSGs and OCGs, we constructed a target profile for each TSG or OCG by examining target genes as being present or absent as related to our hierarchical regulatory network. For a given TSG or OCG, if there is a regulatory relationship between the TSG/OCG and one target gene, the assigned value for the target gene of the TSG/OCG would be one; otherwise, it would be assigned a value of zero. Thus, for a given TSG/OCG, a target profile includes a string with 65 entries with 0 or 1. The same procedure was applied to construct a TF profile for each TSG or OCG with 15 entries of 0 and 1. To investigate the regulatory patterns of TSGs and OCGs, hierarchical cluster analyses were conducted on both the target gene profile and TF profile using R package 2.13.2 [37].

### Constructing Regulatory Subnetworks on Apoptosis, Cell Cycle, Hormone Stimulation and Reproduction

To obtain a further understanding of specific functional modules in our regulatory network, we focused on four biological processes, which included apoptosis, cell cycle, hormone response, and reproduction, since they have been reported to play important roles in OVC and were also enriched in our regulatory network [41], [42], [43]. We compiled four functional term lists on apoptosis, cell cycle, hormone response, and reproduction using Gene Ontology (GO), Kyoto Encyclopedia of Genes and Genomes (KEGG) and SwissProt annotation terms from the DAVID online tool [44]. Finally, based on the curated functional terms, we collected 51 (apoptosis), 47 (cell cycle), 21 (hormone response), and 16 (reproduction) OVC genes in our hierarchical regulatory network.

For the above four sets of collected genes, most of them could be mapped to the top and bottom layers in our hierarchical regulatory network. To extract the subnetwork for each process, we first mapped the collected genes to the bottom layer, then found the TFs (middle layer) that linked to these target genes, and finally recruited the process-related TSGs and OCGs (top layer) with links to these TFs. Utilizing this process, we obtained four subnetworks.

### Statistical Tests for Enriched Functional GO Terms and Biological Pathways

To assess the function of the interesting gene sets, we conducted functional enrichment tests using the online tool DAVID [44]. We selected those GO terms or pathways with an adjusted *P*-value less than 0.05 as calculated by the hypergeometric test followed by the Benjamini-Hochberg method [45], which was implemented in the DAVID tool.

To evaluate functional significance of the 112 genes in our final subnetwork, we randomly selected 112 genes from 1257 OVC genes and compare their corrected *P-*value distribution. In total, we performed the randomization ten times and annotated each gene list using DAVID. Based on the Benjamini-Hochberg adjusted *P-*value, we compared the *P-*value distribution for our 112 genes and ten randomly selected gene lists (Figure S6).

## Results

### Overview of the Computational Pipeline to Construct a Hierarchical Regulatory Network

**Figure 1:**
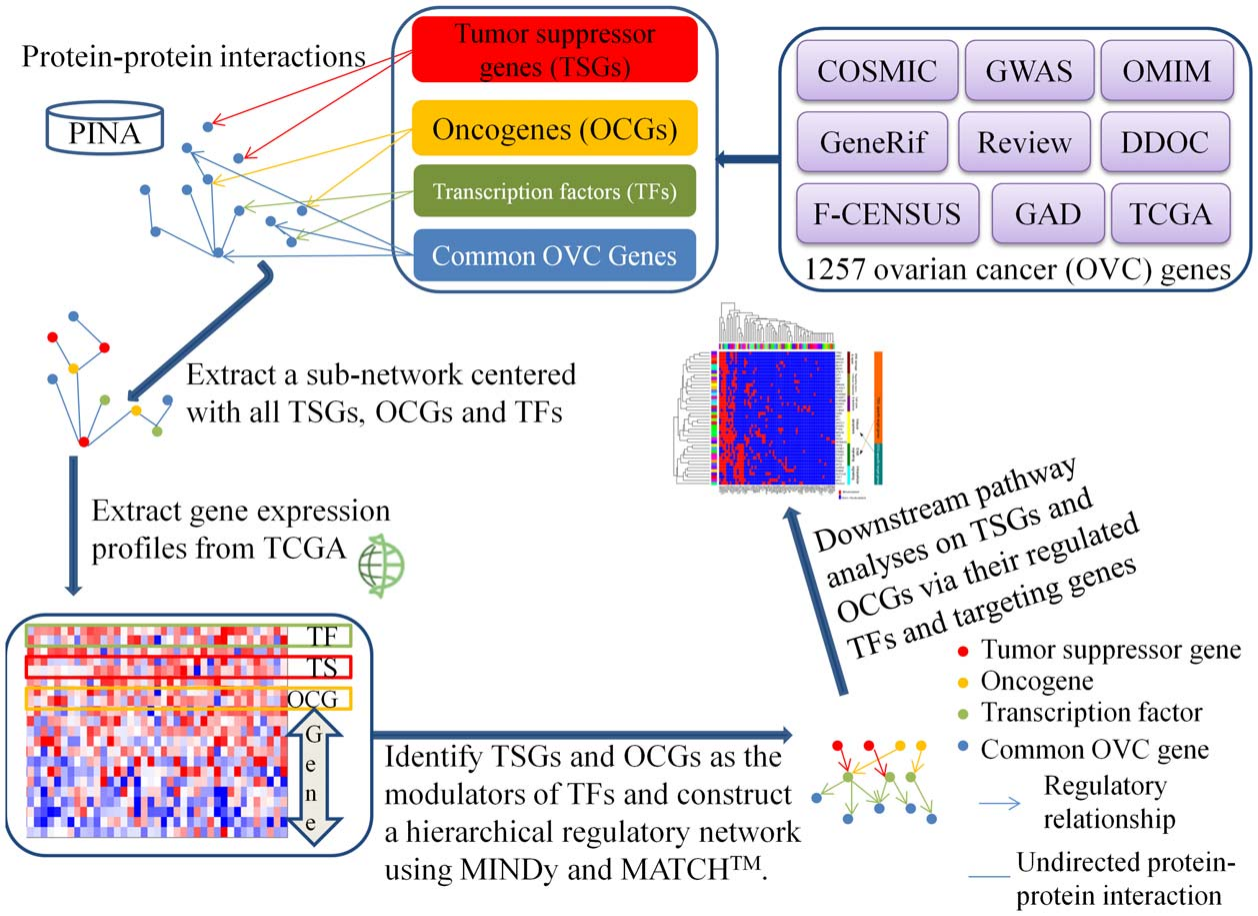
Schematic view of tumor suppressor genes (TSGs) and oncogenes (OCGs) regulatory network analysis. This figure shows the TSG and OCG regulatory network construction and identification of critical downstream pathways modulated by TSGs and OCGs. Our pipeline involves four main steps. 1) Collecting ovarian cancer (OVC)-related genes, tumor suppressors (TSGs), oncogenes (OCGs), and transcription factors (TFs) from public databases and literature. 2) Extracting subnetworks centered on OVC TSGs, OCGs, and TFs from protein-protein interaction (PPI) data. 3) Integrating genome-scale expression data to construct a hierarchical regulatory network with OVC-related TSGs, OCGs, TFs and target genes. 4) Analyzing downstream pathways and subnetworks with regulated genes to investigate the interplay of TSGs and OCGs in specific biological processes. Modulator Inference by Network Dynamics (MINDy) is a software tool used for the identification of post-translational modulators of TFs based on expression profiles. Protein Interaction Network Analysis (PINA) is a platform for protein interaction network construction.

As shown in Figure 1, our pipeline started with a compilation of candidate genes, TSGs, OCGs, and TFs for a certain cancer, such as ovarian cancer. Next, we extracted a subnetwork centered with these known TSGs, OCGs, TFs, and their direct interactors from the human PPI network. Then, gene expression profiles for the genes involved in the subnetwork were inputted into MINDy to infer TSGs and OCGs as post-translational modulators of TFs. The output of MINDy was a regulatory network that comprised the top layer with modulators (TSGs and OCGs), the middle layer with TFs, and the bottom layer with joint target genes of the two top layers. To further reduce the false positives of TF-target relationships inferred from MINDy, we required that the predicted TF-target pairs were also confirmed by the TF-target prediction tool MATCH™. The final output of this pipeline was a combinatory regulatory network of TSGs, OCGs, their modulating TFs, and regulating target genes. We applied this computational pipeline to our curated 1257 OVC candidate genes and, finally, constructed a three-layer regulatory network that included 29 TSGs, 13 OCGs, 15 TF genes, and 65 joint target genes.

### TSGs, OCGs and TFs in OVC Show High Connectivity, Betweenness and Closeness Centrality in the Human PPI Network

Based on the comparison of the three topological properties (degree, betweenness centrality, and closeness centrality) for five cancer-related gene lists, we gained the first insights into the architecture of OVC-related genes in human PPIs (see Materials and Methods). Figure S1 displays the degree distribution of the five datasets and all the proteins in the human PPI network. The average degree of the 97 genes (OVC TSGs, OCGs and TFs) was 56.12, which was significantly higher than that of genes from the cancer gene census (35.33, Kolmogorov-Smirnov test (KS test), *P*-value = 3.36×10^−2^) or that of 905 OVC common genes (21.83, KS test *P*-value = 7.12×10^−8^). To evaluate the significance of the calculated topological properties, we applied an empirical re-sampling approach to compute empirical *P-*values for each properties (Table S6). Except the *P-*value of betweenness centrality from TFs (0.08), all the remaining *P-*values were less than 0.01. These empirical *P*-values suggested that the observed network features were unlikely generated by chance. Further KS tests showed that the betweenness and closeness centralities of the 97 genes were all significantly higher than those genes from the Cancer Gene Census and the 905 OVC common genes (*P*-values <0.05, Table S2). Our comparison between 97 genes (OVC TSGs, OCGs, and TFs) and other cancer genes implied that these 97 genes had higher local connections and shorter paths to other proteins. Given their high degree, betweenness and closeness centralities, a subnetwork with their direct interactors might be enough to characterize the regulatory properties of the 97 genes.

### An OVC-specific Regulatory Gene Network Modulated by TSGs and OCGs

**Figure 2:**
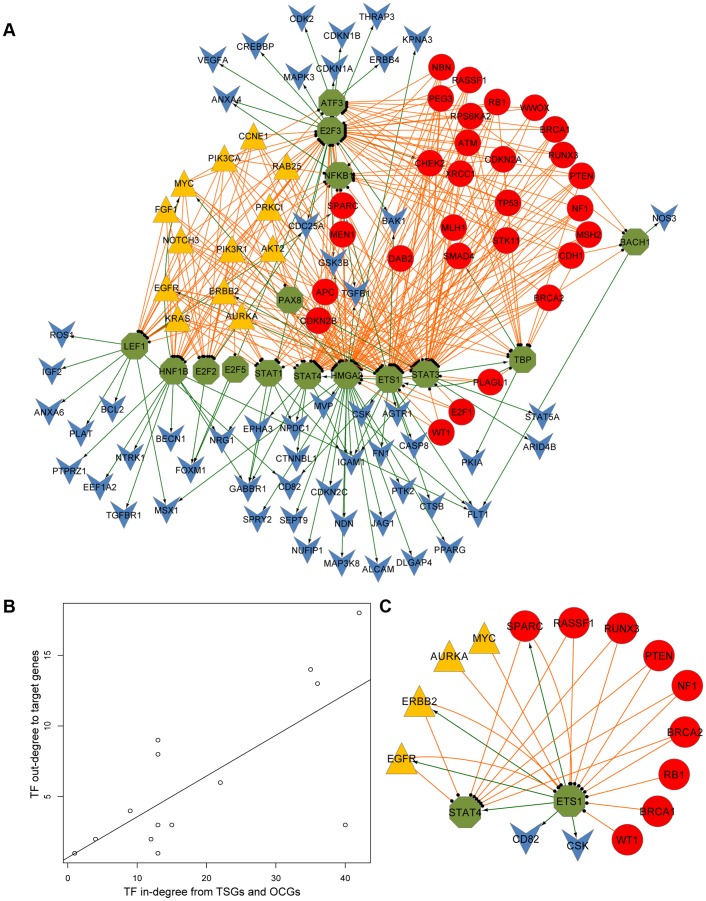
Network view of tumor suppressor genes (TSGs) and oncogenes (OCGs) in ovarian cancer. (A) Integrated hierarchical network of ovarian cancer (OVC) related tumor suppressor genes (TSGs), oncogenes (OCGs), and transcription factors (TFs). The nodes in red (circle) represent OVC-related TSGs, nodes in yellow (triangle) represent OVC-related OCGs, nodes in green (octagon) represent OVC-related TFs, and nodes in blue (vee) represent target genes. The links in orange represent the regulations from the TSGs or OCGs to their modulating TFs. The green arrow lines represent the regulations from the TFs to their target genes. (B) Plot of in-degree and out-degree of the 15 TFs in the three-layer regulatory network. In-degree is defined as the number of nodes that immediately link to and regulate the node of interest, and out-degree is defined as the number of nodes that immediately link to and are regulated by the node of interest. (C) A subnetwork with three feedback loops centered by ETS1. The color and shape schema of nodes and links are the same as those in (A).

Starting from the 97 genes, we integrated the PPIs, gene expression profiles, and TF-target prediction data from the softwares MINDy and MATCH™ (see Materials and Methods). A final three-layer regulatory network was constructed that included 112 unique genes (29 TSGs, 13 OCGs, 15 TF genes and 65 joint target genes) and 353 links (Figure 2A and Table S3). Functional enrichment analyses showed that the 112 genes were enriched in numerous functional categories related to carcinogenesis (Table S4). The most enriched functional categories were related to OVC progression, such as “regulation of apoptosis” (adjusted *P*-value = 4.84×10^−24^), “regulation of cell cycle” (adjusted *P*-value = 8.10×10^−20^), “response to hormone stimulus” (adjusted *P*-value = 5.31×10^−10^), and “multicellular organism reproduction” (adjusted *P*-value = 1.06×10^−4^). To evaluate the functional analysis results of the 112 genes, we randomly selected ten gene lists with 112 genes from 1257 OVC genes. As shown in the Figure S6, most of corrected *P-*values from functional terms annotated for our 112 genes were less than 0.01, which is distinctly different from the ten randomly selected gene lists (Kolmogorov-Smirnov test, *P-*values <0.05). These highly enriched carcinogenesis-related functions demonstrated that our regulatory network was closely related to OVC development and might be useful to identify core cancerogenesis modules.

As shown in Figure 2B, the TFs’ in-degrees from TSGs and OCGs had a high correlation with their out-degrees to their target genes (Pearson’s correlation coefficient = 0.74, *P*-value = 1.60×10^−3^). Here, in-degree is defined as the number of TSG/OCG nodes that immediately link to and regulate the TF node, and out-degree is defined as the number of target gene nodes that immediately link to and are regulated by the TF node. For instance, the in-degree and out-degree of TF ETS1 were 34 and 14, respectively; that is, it was modulated by the 23 TSGs and 11 OCGs while it regulated 14 downstream target genes. Additionally, the 14 target genes of ETS1 were enriched in “tyrosine protein kinase” based on GO annotation (adjusted *P*-values = 2.15×10^−3^). Overall, our results suggest that TFs with more inputs in our regulatory network could regulate more target genes.

In this network, all regulatory signals from TSGs and OCGs were passed to the middle layer (populated by TFs) and then transferred to the bottom layer with target genes. However, 10 of the 65 target genes also belong to TSGs, OCGs or TF genes in our network. The 10 genes with multiple roles formed six regulatory loops between TFs and TSGs/OCGs. The loops were E2F3 ↔ CHEK2, ETS1 ↔ EGFR, ETS1 ↔ ERBB2, ETS1 ↔ SPARC, HMGA2 ↔ MYC, and HNF1B ↔ MYC (Table S5). For example, ETS1 formed three feedback loops with genes encoding EGFR, ERBB2, and SPARC. Among the three genes, EGFR and ERBB2 belong to the epidermal growth factor receptor (EGFR) family (Figure 2C). We further recruited direct interactors of ETS1 related to the three feedback loops to form a subnetwork. There were a total of 17 genes in the subnetwork specific to ETS1, which were enriched with the GO biological processes terms “regulation of cell proliferation” (adjusted *P*-value = 1.94×10^−8^) and “regulation of cell cycle” (adjusted *P*-value = 9.77×10^−6^). These analyses indicate that ETS1 might play important roles in cell proliferation through its interactions with the EGFR family, which is consistent with the results from previous studies of human cancer cells [46].

### TSGs and OCGs were Involved in different Biological Processes in OVC

To further identify the specific biological processes in which TSGs and OCGs participate, we performed functional enrichment analyses for TSGs and OCGs independently. The results indicated that TSGs and OCGs were involved in different biological processes during the carcinogenesis of OVC (Table 1). The 29 TSGs were mainly involved in cell cycle, DNA damage response, positive regulation of apoptosis, and the negatively regulating macromolecule metabolic process, whereas 13 OCGs were mainly involved in signaling pathways, such as ErbB and response to hormone stimulus, and, opposite to the TSG results, negative regulation of apoptosis. Among the 112 genes in our network, 46 genes were mapped to the KEGG “pathway in cancer” (13 TSGs, 9 OCGs, 8 TF genes and 16 target genes) (Figure S3). The map showed most of TSGs and OCGs regulated important carcinogenesis processes. The majority of TSGs surrounded processes like cell cycle, DNA repair and apoptosis; OCGs tended to interact with the signaling input, while TF genes and target genes often scattered across the map to connect TSGs and OCGs.

**Table 1:** Gene Ontology (GO) terms and KEGG pathways overrepresented in tumor suppressor genes (TSGs), oncogenes (OCGs), TSG-specific target genes, and OCG-specific target genes.

Biological function	Adjusted *P-*value^a^
**29 tumor suppressor genes (TSGs)**	
GO: cell cycle	1.04×10^−10^
GO: positive regulation of apoptosis	2.58×10^−6^
GO: DNA damage response, signal transduction	3.08×10^−6^
GO: negative regulation of macromolecule metabolic process	5.81×10^−6^
GO: regulation of cell proliferation	8.80×10^−6^
**13 oncogenes (OCGs)**	
KEGG: ErbB signaling pathway	2.59×10^−7^
GO: response to organic substance	1.46×10^−5^
GO: intracellular signaling cascade	2.42×10^−5^
GO: response to hormone stimulus	5.85×10^−5^
GO: negative regulation of apoptosis	9.25×10^−4^
**16 TSG-specific target genes**	
KEGG: ErbB signaling pathway	9.31×10^−4^
**14 OCG-specific target genes**	
GO: enzyme linked receptor protein signaling pathway	8.01×10^−6^
GO: negative regulation of apoptosis	5.09×10^−6^
GO: transmembrane receptor protein tyrosine kinase signaling pathway	5.15×10^−6^
GO: positive regulation of growth	4.2×10^−3^
GO: regulation of growth	0.016

The functional enrichment test was performed by the DAVID tool [44]. For a gene set that had more than five functional terms with an adjusted *P*-value of less than 0.05, the top five terms were listed in this table.

a Adjusted *P*-values: the *P*-values of the hypergeometric test were corrected by Benjamini-Hochberg multiple testing correction [45].

Previous studies have shown that TSGs are involved in tumorigenesis because their products are generally involved in cell cycle checkpoint, apoptosis, or the repair of damaged DNA [2], and OCGs encode chromatin remodelers, growth factors and receptors, signal transducers, and apoptosis regulators [3]. Our results of functional analyses of OVC TSGs and OCGs were consistent with their common roles in tumorigenesis. It is worth noting that our analyses indicated that the OCGs had important roles in the process “response hormone signals,” which is important to accelerate cell proliferation during OVC development, including gonadotropins, estrogens, androgens, progesterone and insulin [18].

### TSGs and OCGs had a Distinct Regulatory Pattern

**Figure 3:**
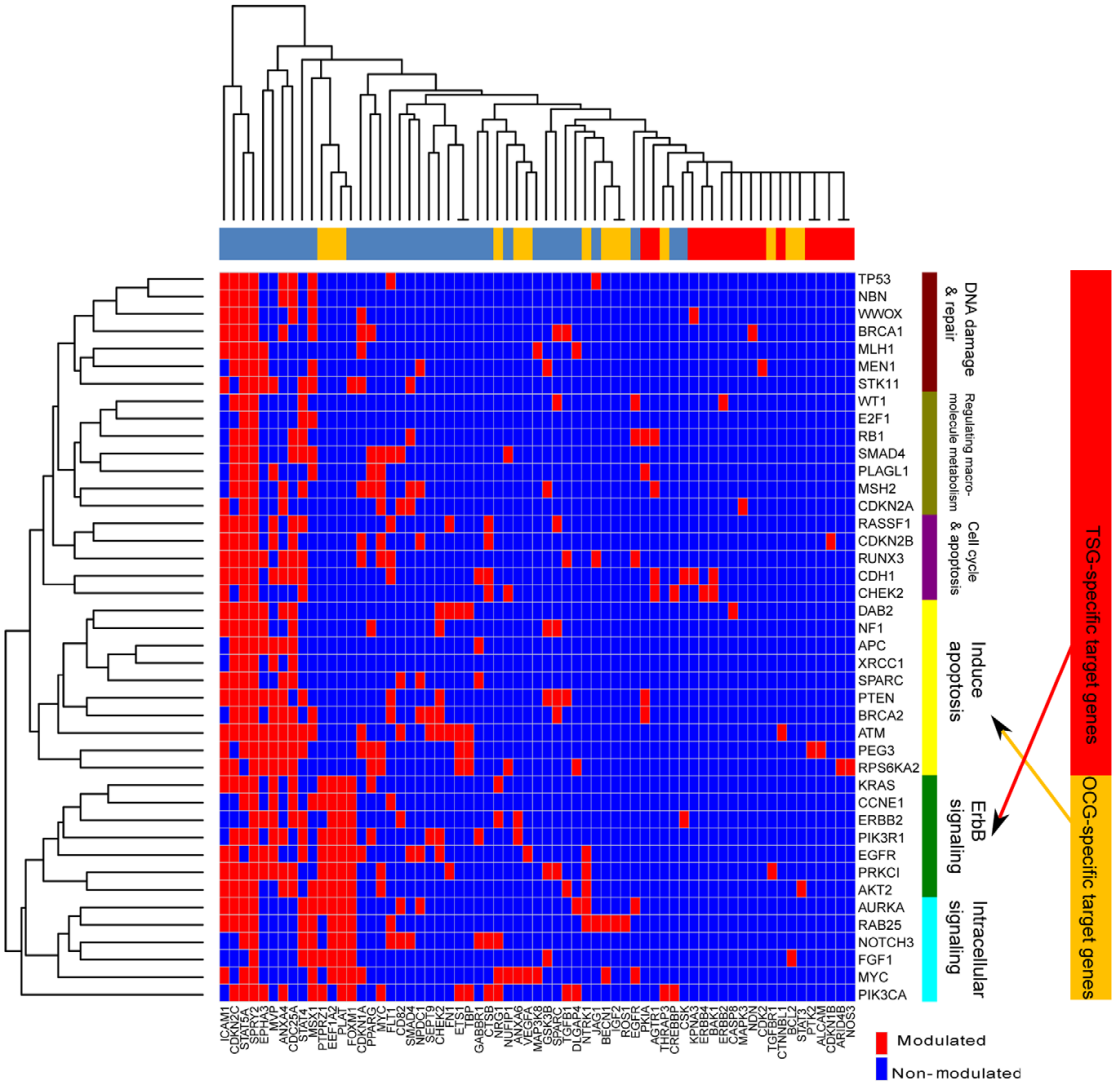
Downstream target gene profiles clustering with tumor suppressor genes (TSGs) and oncogenes (OCGs). The heat map shows a two-color representation of the regulatory relationship between modulators (TSGs and OCGs) and downstream target genes. A red colored cell in the grid indicates that the row TSG or OCG is inferred to regulate the column target gene. A blue colored cell in the grid indicates that the row TSG or OCG has no influence on the column target gene. The modulators’ dendrogram represents a hierarchical clustering of TSGs and OCGs based on their target gene profiles. The modulators’ dendrogram is divided into two branches with six clusters marked with different colors. The most significant enriched functional annotations are marked along the right of each cluster. Take the first maroon cluster in the TSG branch as an example: the enriched genes are involved in DNA damage and repair. The TSG-specific target genes are marked in red and the OCG-specific target genes are marked with yellow in the top panel. In addition, the TSG-specific target genes are also represented in red and the OCG-specific target genes are represented as a whole with yellow in the right panel. The arrow from TSG-specific target genes represents their regulatory effects on the ErbB signaling pathway, and the arrow from OCG-specific target genes represents their anti-apoptosis effects as apoptosis negative regulators.

To discover the difference in regulatory patterns between TSGs and OCGs, we constructed two groups of regulatory profiles for the 42 modulators (29 TSGs and 13 OCGs): one was based on 15 TFs that were either directly regulated or not regulated by TSGs or OCGs, while the other was based on 65 target genes that were either indirectly regulated or not regulated by TSGs or OCGs through TFs (see Material and Methods). Based on these profiles, hierarchical clustering analyses were performed. Figure S2 shows the clustering results based on TFs' profiles, and Figure 3 shows the clustering results based on target genes' profiles. The two clustering results consistently demonstrated that the TFs' 42 modulators were clustered into two distinct branches: one branch contained all 29 TSGs (TS branch), and the other branch included all 13 OCGs (OCG branch). These observations indicated that TSGs and OCGs might play distinct roles in OVC progression, which is consistent with the above observation that they were involved in different biological processes.

Since target genes might play important roles in the generation of the phenotypes, we further performed functional enrichment analyses of TSGs or OCGs in sub-groups from target gene-based clustering analysis (Figure 3). There were four clusters in the TSG branch and two clusters in the OCG branch. In the first TSG cluster, the seven genes were enriched with the GO biological process term “DNA repair” (adjusted *P*-value = 4.42×10^−4^). In fact, besides the five TSGs (TP53, NBN, MLH1, MEN1, and BRCA1) annotated with DNA repair by GO annotations, the remaining STK11 was related to “response to DNA damage stimulus” based on GO annotation, and WWOX was also reportedly involved in DNA damage during carcinogenesis [47]. Therefore, all seven TSGs in the first TSG cluster might play roles in DNA damage and repair. The seven TSGs in the second cluster mainly regulate macromolecular metabolism because of their enrichment in the GO biological process terms “negative regulation of macromolecule metabolic process” (adjusted *P*-value = 0.0011) and “positive regulation of transcription from RNA polymerase II promoter” (adjusted *P*-value = 0.0015). The third TSG cluster was comprised of six genes, three of which (*CDKN2B*, *CHEK2*, and *RASSF1*) were related to cell cycle and two of which (*CHEK2* and *RUNX3*) could induce apoptosis according to GO annotations. Thus, the cluster might be related to cell cycle and apoptosis. In the fourth TSG cluster, ten TSGs were enriched with “positive regulation of programmed cell death” (adjusted *P*-value = 0.0299). Among the ten genes, five of the TSGs (*ATM*, *APC*, *BRCA2*, *NF1*, and *PTEN*) were annotated with positive effects on apoptosis; the other three TSGs (*PEG3*, *SPARC*, and *RPS6KA2*) were also reported to increase apoptosis in sporadic epithelial OVC or other types of cancer [48], [49], [50]. Therefore, the TSGs from the fourth cluster were grouped together, as they might function in “induction of apoptosis.”

In the OCG branch, the first cluster contained seven OCGs, which were enriched in the GO biological processes “ErbB signaling pathway” (adjusted *P*-value = 1.24×10^−5^) and “response to hormone stimulus” (adjusted *P*- value = 1.19×10^−4^). For the second cluster containing six OCG genes, there were no significant biological functions observed. However, four of the six (*AURKA*, *FGF1*, *PIK3CA*, and *RAB25*) were annotated with “intracellular signaling cascade” by GO analysis. Thus, the cluster might be related to intracellular signaling. These observations indicated that the TSGs in OVC were mainly related to fundamental cell growth processes, such as cell cycle, apoptosis, and DNA damage and repair, while the OCGs were more specific to response signaling, including ErbB.

**Figure 4:**
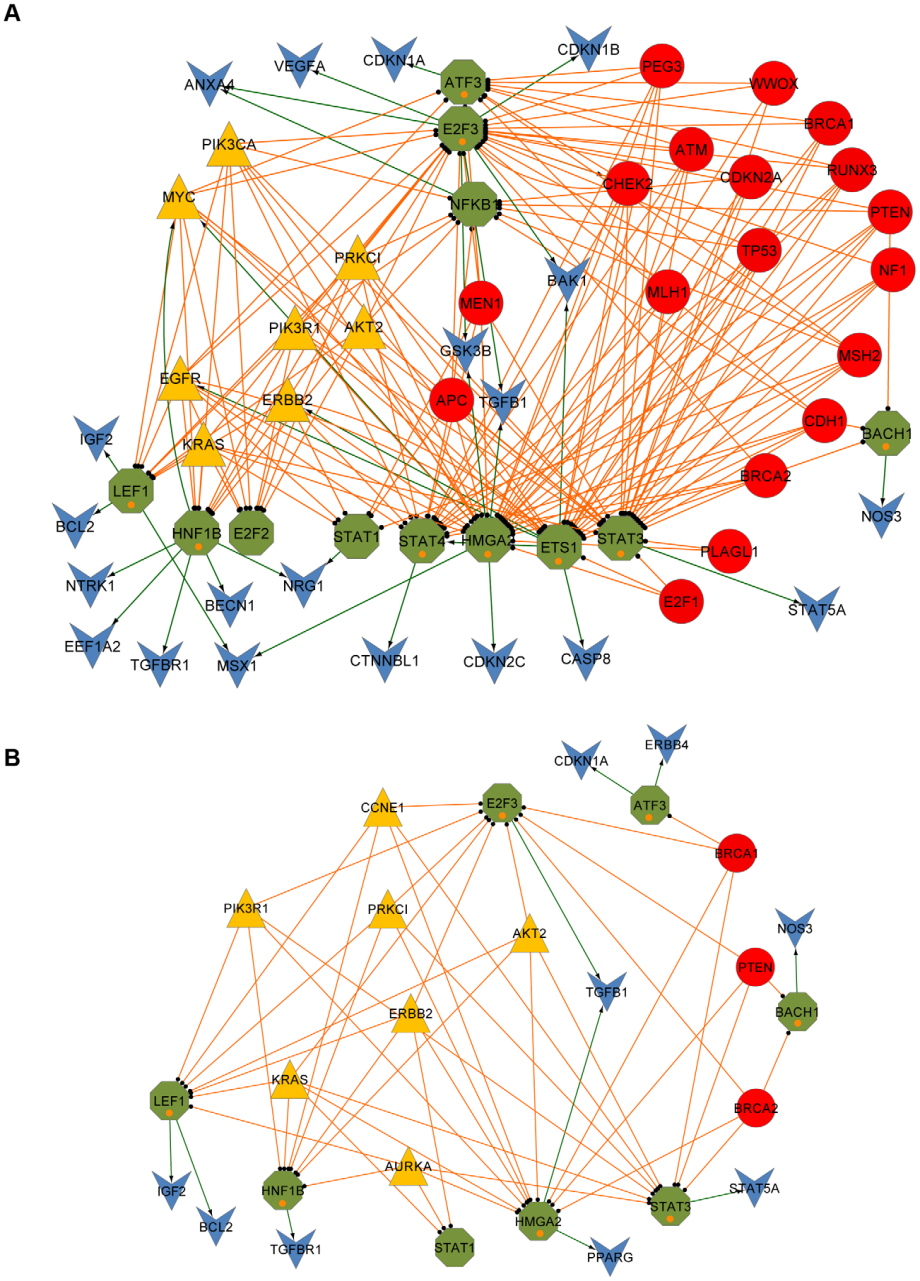
Interplay of tumor suppressor genes (TSGs) and oncogenes (OCGs) to regulate apoptosis and response to hormone stimulation. (A) Apoptosis. (B) Response to hormone stimulation. The red circular nodes are OVC-related TSGs. The yellow triangle shaped nodes are OVC-related OCGs. The green octagonal nodes are OVC-related transcription factors (TFs). The blue vee nodes represent targeted OVC genes. The orange links are from the TSGs or OCGs to their modulating TFs. The green arrow lines are from the TFs to their regulating target genes. The TFs added by the first neighbors of the target genes involved in the two biological processes are marked with orange circles in (A) and (B).

Interestingly, target genes specifically regulated by TSGs and OCGs showed competitively regulatory patterns in two biological processes compared to their respective regulators (OCGs and TSGs) (Figure 3). The first one was observed in the ErbB signaling pathway. Although 29 TSGs mainly focused on DNA damage and repair, regulating macromolecular metabolism, cell cycle, apoptosis, and induced apoptosis, we observed that TSGs' specific target genes were enriched in the ErbB signaling pathway (adjusted *P*-value = 9.31×10^−4^). This pathway was found to be enriched in the 13 OCGs (Table 1) and the first cluster in the OCG branch (Figure 3). Another competitively regulatory pattern was observed in apoptosis. Among the 14 target genes that were regulated by OCGs only, negative regulators on apoptosis were found to be statistically enriched (adjusted *P*-value = 5.09×10^−6^) (Table 1). Taking together the six OCGs (*EGFR*, *KRAS*, *PRKCI*, *ERBB2*, *PIK3CA*, and *MYC*) as negative apoptosis regulators, there were 14 negative regulators of apoptosis directly or indirectly related to OCGs in our network (Figure 2A). In contrast, 14 TSGs and four TSG-specific target genes were positive regulators of apoptosis. Therefore, in the apoptosis subnetwork (Figure 4A), OCGs and their specific targets have roles in the prevention of apoptosis, whereas TSGs and their specific targets tend to promote apoptosis. The results from this unique regulatory network analysis revealed that, although some TSGs were involved in apoptosis and OCGs were mainly involved in ErbB signaling (Figure 3), their specific target genes might reverse the process functions in apoptosis and the ErbB signaling pathway.

### Interplay of TSGs, OCGs and their Modulating TFs to Regulate Apoptosis, Cell Cycle, Reproduction and Response to Hormone Stimulation

To better understand the molecular activities in the important biological processes in OVC, we generated and analyzed four subnetworks related to “apoptosis,” “response to hormone stimulus,” “cell cycle,” and “reproduction” (see Materials and Methods). For this purpose, we filled the regulatory gaps between TSGs/OCGs (top layer) and target genes (bottom layer) with TFs (middle layer) in each subnetwork if those TFs directly regulated the target genes related to each biological process. In the apoptosis subnetwork, there are 17 TSGs, 8 OCGs, 12 TF genes, and 17 target genes. Among the 12 TFs, nine were added as direct regulators of target genes in the apoptosis subnetwork (Figure 4A). For the nine added TF genes, seven of them were reported to take part in apoptosis in various types of cancer or diseases; these seven TFs are STAT3 [51], [52], [53], ETS1 [54], LEF1 [55], [56], STAT4 [57], E2F3 [58], ATF3 [59], and HMGA2 [60], which have not been annotated by GO yet. Notably, HMGA2 demonstrated its function to increase apoptosis in OVC [60]. Similarly, in the hormone stimulus subnetwork (Figure 4B), four added TFs, ATF3 [61], E2F3 [62], HMGA2 [63], [64], and LEF1 [65], were reported to respond to hormone stimuli. For the cell cycle subnetwork (Figure S4A), three added TFs (STAT3, ATF3, and TBP) were confirmed by previous studies [66], [67], [68]. In the reproduction-related subnetwork (Figure S4B), three added TFs, STAT3, BACH1, and LEF1, had evidence that they were related to the reproductive system [69], [70], [71]. In total, 26 added TFs were from the four subnetworks, and 17 TFs (65.38%) had literature evidence to support the novel functions in their corresponding subnetworks.

The majority of TSGs and OCGs interplayed in multiple related cellular processes, and they tended to co-regulate in close relation to each other in those processes. In the cell cycle subnetwork, 65% of TSGs, 50% of OCGs and 87.5% of TFs were shared with those from the apoptosis subnetwork (Figures S5A, B, and C). For the 22 genes in the reproduction subnetwork, 14 were shared with 27 genes in the hormone stimulation subnetwork (Figures S5A, B, and C). Therefore, TSGs and OCGs could co-regulate cell growth related processes in opposite ways, such as in cell cycle and apoptosis. Additionally, both TSGs and OCGs might play important roles regarding the response to hormone stimuli and reproduction.

## Discussion

In this study, we explored the hierarchical regulatory networks involving tumor suppressor genes, oncogenes, and transcription factors in conjunction with the human protein interactome and gene expression profiles. We developed a computational framework for construction and analysis of the hierarchical regulatory networks and successfully demonstrated it in a major type of cancer, ovarian cancer. The key aspect of our approach was the utilization of TFs as bridges to link the regulatory impact of TSGs and OCGs to target genes, which was expected to address the inherent indirect association of TSGs and OCGs to downstream target genes. Our framework started with the extraction of a PPI subnetwork centered with modulators (TSGs, OCGs and TFs), and we then inferred regulatory relationships using gene expression profiles. Based on these relationships, we constructed a three-layer regulatory network that included TSGs, OCGs, TFs, and their joint target genes. This general network analysis pipeline is likely robust to recruit critical genes and their regulation in tumorigenesis and can be applied to discover the regulatory relationship between TSGs, OCGs, TFs, and target genes in other types of cancer or other complex diseases. However, this process involved multiple computational methods, such as clustering, functional enrichment analysis of a gene set, and definition of gene sets. Thus, as in many complicated computational analyses, one should be cautious when interpreting the results.

Through the construction and analyses of the regulatory networks and subnetworks, and functional enrichment analyses of TSGs, OCGs, TFs, and their target genes, we observed that TSGs could play functional roles in DNA damage and repair, cell cycle, apoptosis, and the regulation of macromolecule metabolism, while OCGs may be primarily involved in ErbB signaling and the response to hormone stimuli. Furthermore, the TSG- and OCG-specific targets showed a reversed functional distribution, i.e., the TSG-specific target genes were enriched in the ErbB signaling pathway, whereas the OCG-specific target genes were involved in apoptosis. Taken together, these results suggested that TSGs might regulate the biological processes that are mainly regulated by OCGs via TSG-specific target genes, while OCGs could participate in the regulation of the biological processes that are mainly regulated by TSGs via OCG-specific target genes. Therefore, there is a competitive regulation mechanism between TSGs and OCGs, which could play an important role during cancer development. Until now, based on our knowledge, there has been no report of any competitive regulation model between these two major types of genes in any type of cancer. Further investigation in other types of cancer or disease is warranted.

Over the past few decades, the identification of disease candidate genes and the investigation of their regulatory mechanisms are important in order to understand the biological processes of disease, including those in cancer [72]. To provide a clearer picture of OVC genetics, we performed a comprehensive review and analysis of published literature and data, resulting in a total of 1257 genes identified as related to OVC. We hope the data presented in this study will be a valuable resource for the OVC research community to explore both TSGs and OCGs in various cancerogenesis processes. We roughly ranked 1257 genes based on the number of data resources (Table S1). Many well-known OVC causal genes, such as *BRCA1*, *BRCA2*, *TP53*, and *PTEN*, were ranked in the top positions. Therefore, the gene list, along with investigators’ own datasets, might be a valuable resource for the OVC research community to further investigate OVC. In addition, the OVC-specific regulatory network generated in this study might be comprised of important regulatory relationships between hub TFs (highly connected TFs in the network), their modulators (Figure 3C), and novel functions of TFs (Figure 4 and S4); these relationships, in turn, could provide interesting clues for further investigation. For instance, a number of feedback regulatory loops are known to synchronize with cell proliferation [73]. It is therefore interesting to observe that three feedback loops centered with ETS1 and the EGFR family contain 12 genes that are able to regulate cell proliferation. Although ETS1 which has functions in stem cell development, cell senescence and death, and tumorigenesis [74] – has important roles in our regulatory network, there are few studies that focus on the roles of ETS1 in OVC [75]. In contrast, the other hub TF HMGA2 was extensively studied recently as a promising biomarker in OVC [60], [76]. Exploring regulatory relationships centered on ETS1 and HMGA2 using targeted experiments may lead to new insight regarding growth signaling networks in cell proliferation of OVC.

## Supporting Information

Figure S1 **Degree distribution in the human protein-protein interaction network.** The empirical cumulative distribution functions (ECDFs) for degrees of different gene datasets. The ECDF curves (black) represent the degree of all the human genes in the protein-protein interaction network: the blue curve represents the degrees of 1257 ovarian cancer (OVC)-related genes; the green curve represents the 467 cancer census genes; the red curves are the degrees of all the involved OVC TSGs, OCGs and TFs; the orange, pink and navy curves represent the degrees of the OVC TSGs, OCGs and TFs, respectively.
(TIF)

Figure S2 **Transcription factor (TF) profile clustering for tumor suppressor genes (TSGs) and oncogenes (OCGs).** The heat map shows a two color representation of the regulatory relationship between modulators and TFs, and the dendrogram represents a hierarchical clustering of modulators and regulated TFs. A red colored cell in the grid indicates that the row TSG or OCG is inferred to regulate the column TF. A blue colored cell in the grid indicates that the row TSG or OCG has no influence on the column TF.
(TIF)

Figure S3 **Interplay of tumor suppressor genes (TSGs) and oncogenes (OCGs) on a cancer pathway annotated by KEGG.** The genes with a red background and blue label are TSG genes; the genes with a brown background and cyan label are OCG genes; the genes with a green background and black label are transcription factor (TF) genes; the genes with a yellow background color and navy label are target genes.
(TIF)

Figure S4 **Interplay of tumor suppressor genes (TSGs) and oncogenes (OCGs) to regulate cell cycle and reproduction.** (A) Cell cycle. (B) Reproduction. The nodes with red circles are tumor suppressor genes (TSGs). The nodes with orange diamond are oncogenes (OCGs). The nodes with green octagon are TFs. The genes with blue vee are target genes. The links with orange color are from the TSGs or OCGs to their modulating TFs. The arrow lines with green color are from the TFs to their target genes. The TFs added by the first neighbors of the target genes involved in the two biological processes are marked with orange circles in (A) and (B).
(TIF)

Figure S5 **Overlap of the genes involved in the hierarchical regulatory subnetworks.** Overlap of all the involved tumor suppressor genes (A), oncogenes (B) and transcription factor genes (C) to regulate apoptosis and cell cycle, response to hormone stimulation, and reproduction sub-networks. The AP on each panel represents the gene contents involved in apoptosis; the CC on each panel refers to the gene content involved in the cell cycle; the HA on each panel is the gene content in response to hormone stimulus; and the RP on each panel represents the gene content involved in reproduction.
(TIF)

Figure S6 **The** ***P-*** **value distribution of functional terms from DAVID for the 112 genes in the ovarian cancer-specific regulatory network and ten gene lists randomly selected from 1257 OVC genes with same number of genes.** The empirical cumulative distribution functions (ECDFs) for *P-*values of different gene datasets. The ECDF curves (black) represent the *P-*value of the 112 genes in the ovarian cancer-specific regulatory network. The other ten curves represent the *P-*value of the112 genes randomly selected from 1257 ovarian cancer (OVC)-related genes. For comparison *P-*values less than 0.05, only the proportions of the *P-*values less than or equal to 0.05 were plotted.
(TIF)

Table S1 **List of the 1257 ovarian cancer (OVC) candidate genes collected in this study.**
(XLSX)

Table S2 **The comparison of network topological characteristics in human protein-protein interaction (PPI) among different gene datasets.** The results of Kolmogorov-Smirnov test on degree, betweenness centrality and closeness centrality among different gene datasets are included.
(DOC)

Table S3 **List of the 112 genes in the ovarian cancer-specific hierarchical regulatory network.**
(XLSX)

Table S4 **Functional annotations of the 112 genes in the ovarian cancer-specific regulatory network.** The functional annotation and statistical enrichment results are obtained from the DAVID functional classification tool.
(XLSX)

Table S5 **List of regulatory loops in the ovarian cancer-specific regulatory network.** The file contains all the TFs and their modulators that may regulate each other in a feedback loop style.
(XLSX)

Table S6 **Empirical** ***P-*** **values of network topological characteristics in human protein-protein interaction (PPI) among different gene datasets.** The results of randomization on degree, betweenness centrality and closeness centrality among different OVC TSGs, OCGs, and TFs are included.
(DOC)

Text S1 **This file includes details of data sources and methods that were used for ovarian cancer (OVC) candidate gene collection and curation.**
(DOC)
