# Supplementary figures and images for: Distinct and Competitive Regulatory Patterns of Tumor Suppressor Genes and Oncogenes in Ovarian Cancer
Source: PLoS One. 2012 Aug 30;7(8):e44175. doi: 10.1371/journal.pone.0044175 (PMC3431336; doi:10.1371/journal.pone.0044175)

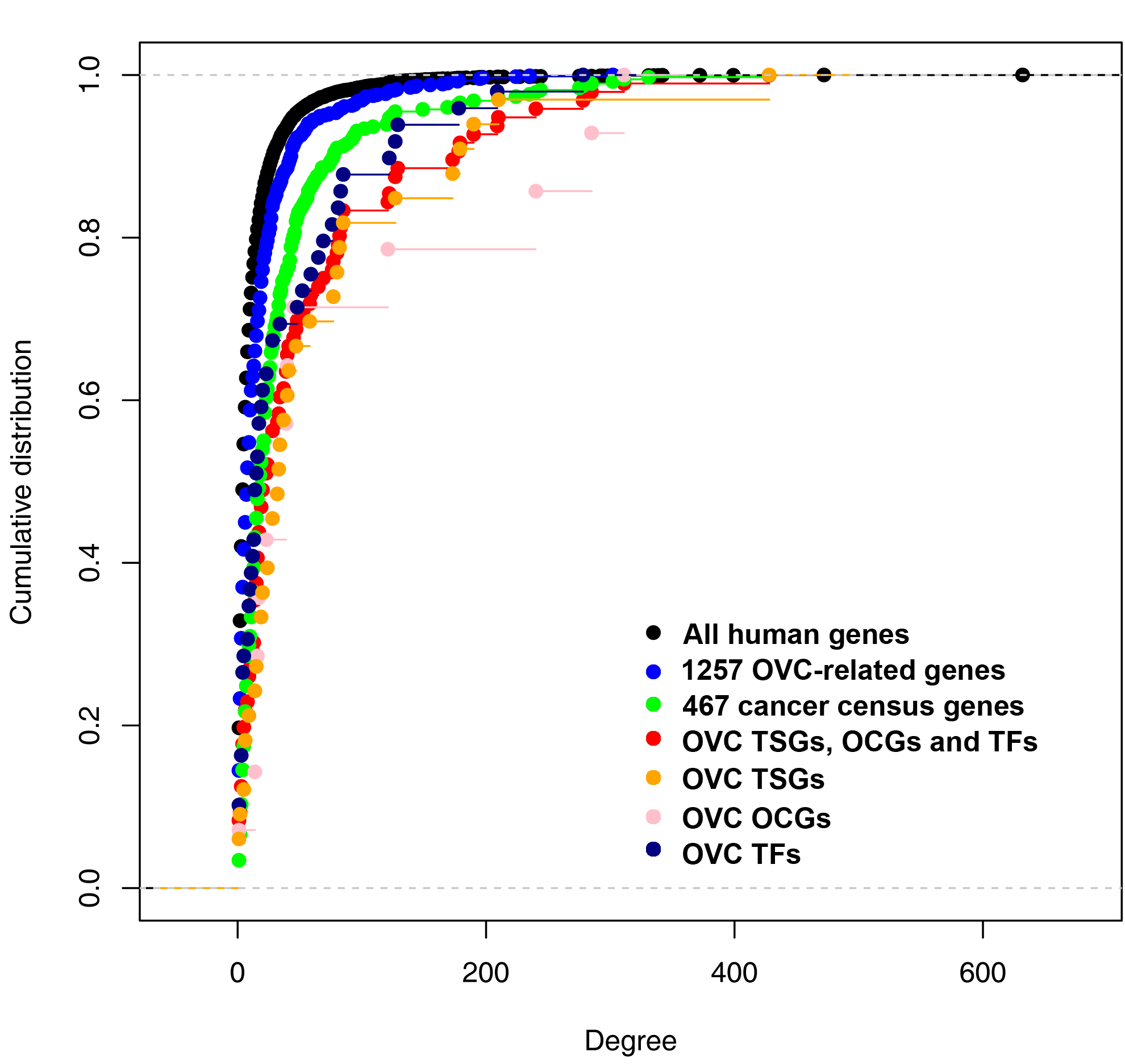

Supplement: Figure S1 — Degree distribution in the human protein-protein interaction network. The empirical cumulative distribution functions (ECDFs) for degrees of different gene datasets. The ECDF curves (black) represent the degree of all the human genes in the protein-protein interaction network: the blue curve represents the degrees of 1257 ovarian cancer (OVC)-related genes; the green curve represents the 467 cancer census genes; the red curves are the degrees of all the involved OVC TSGs, OCGs and TFs; the orange, pink and navy curves represent the degrees of the OVC TSGs, OCGs and TFs, respectively. (TIF) [file pone.0044175.s001.tif]

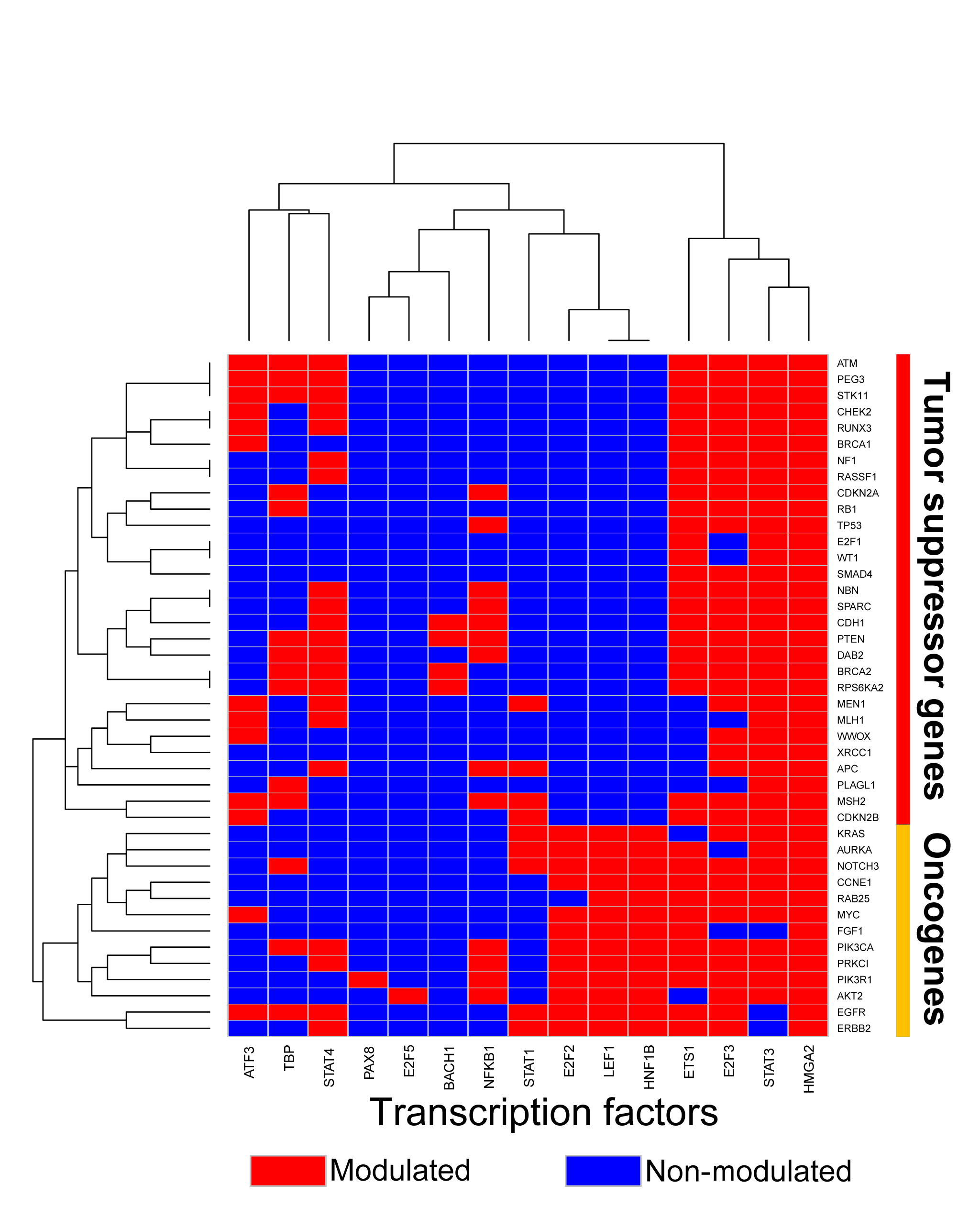

Supplement: Figure S2 — Transcription factor (TF) profile clustering for tumor suppressor genes (TSGs) and oncogenes (OCGs). The heat map shows a two color representation of the regulatory relationship between modulators and TFs, and the dendrogram represents a hierarchical clustering of modulators and regulated TFs. A red colored cell in the grid indicates that the row TSG or OCG is inferred to regulate the column TF. A blue colored cell in the grid indicates that the row TSG or OCG has no influence on the column TF. (TIF) [file pone.0044175.s002.tif]

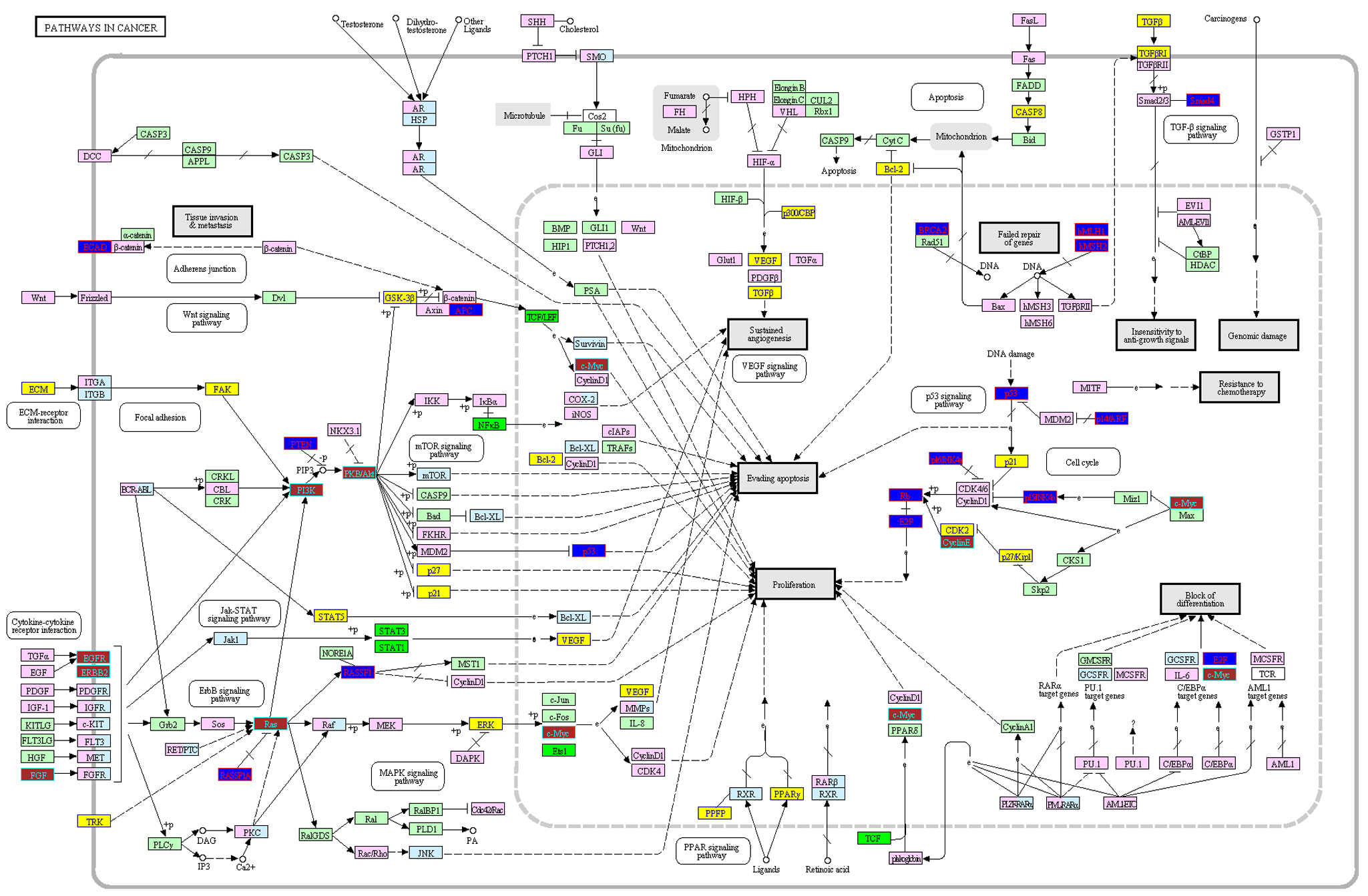

Supplement: Figure S3 — Interplay of tumor suppressor genes (TSGs) and oncogenes (OCGs) on a cancer pathway annotated by KEGG. The genes with a red background and blue label are TSG genes; the genes with a brown background and cyan label are OCG genes; the genes with a green background and black label are transcription factor (TF) genes; the genes with a yellow background color and navy label are target genes. (TIF) [file pone.0044175.s003.tif]

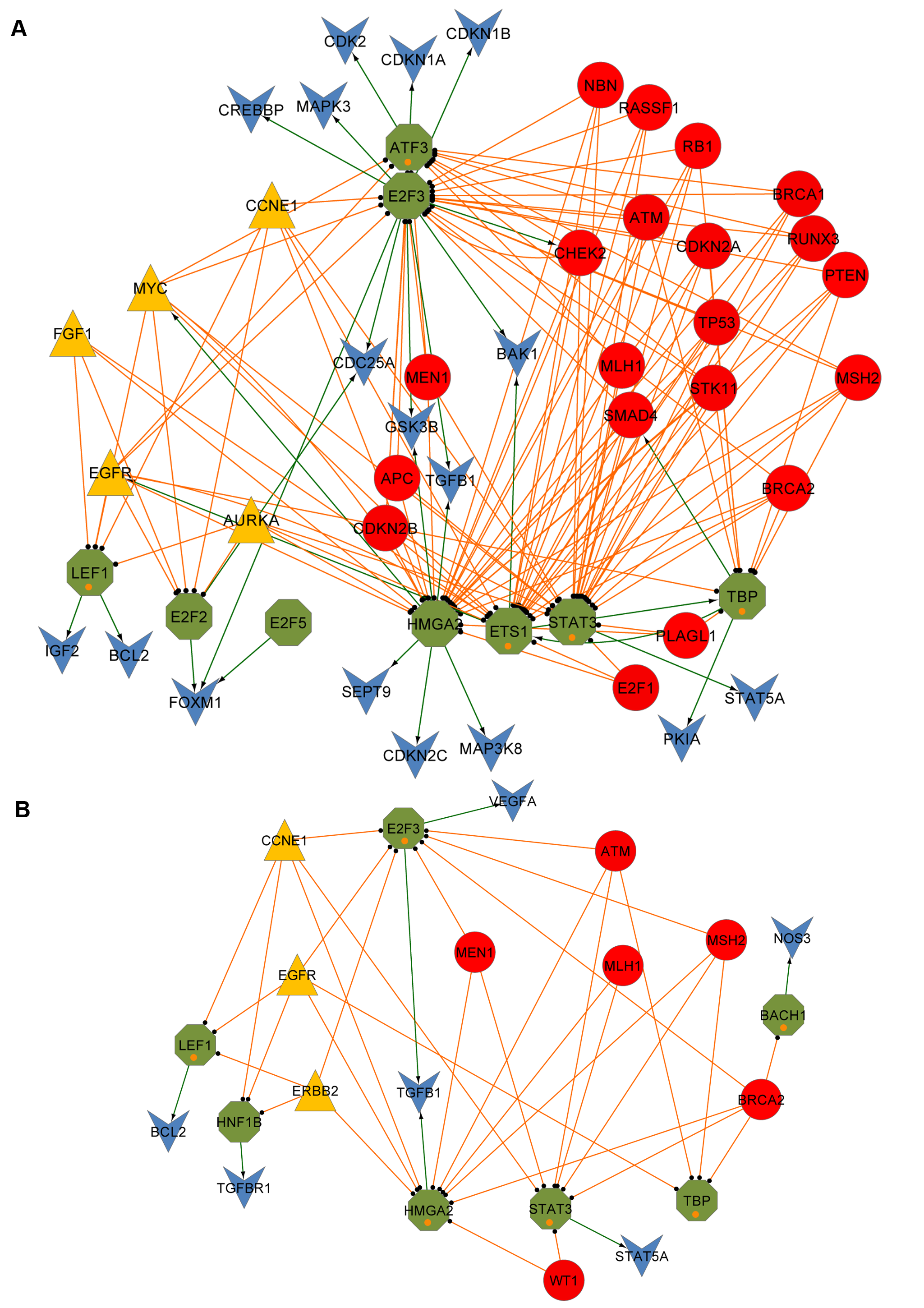

Supplement: Figure S4 — Interplay of tumor suppressor genes (TSGs) and oncogenes (OCGs) to regulate cell cycle and reproduction. (A) Cell cycle. (B) Reproduction. The nodes with red circles are tumor suppressor genes (TSGs). The nodes with orange diamond are oncogenes (OCGs). The nodes with green octagon are TFs. The genes with blue vee are target genes. The links with orange color are from the TSGs or OCGs to their modulating TFs. The arrow lines with green color are from the TFs to their target genes. The TFs added by the first neighbors of the target genes involved in the two biological processes are marked with orange circles in (A) and (B). (TIF) [file pone.0044175.s004.tif]

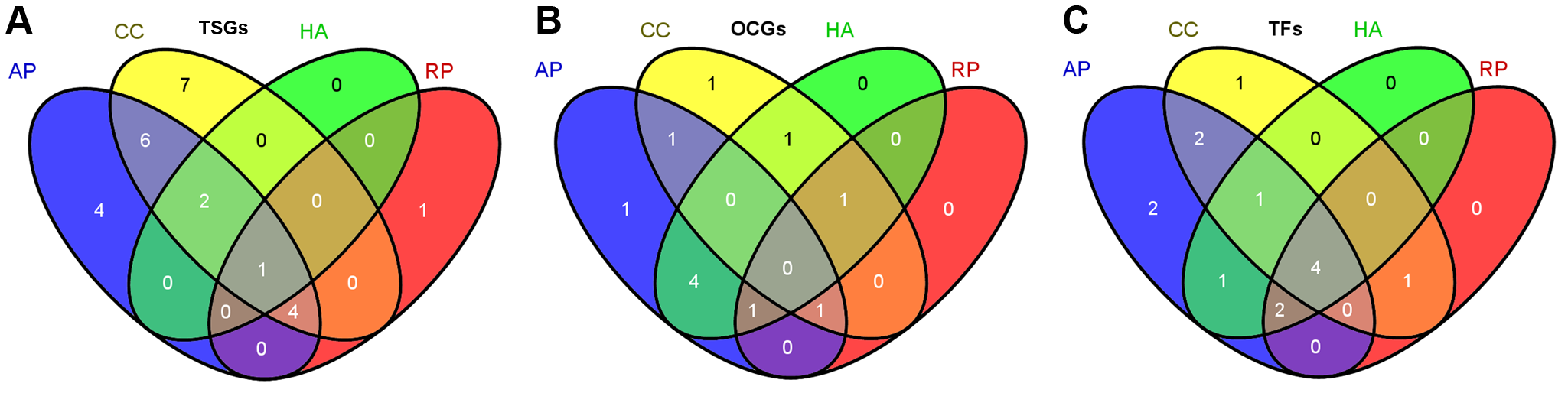

Supplement: Figure S5 — Overlap of the genes involved in the hierarchical regulatory subnetworks. Overlap of all the involved tumor suppressor genes (A), oncogenes (B) and transcription factor genes (C) to regulate apoptosis and cell cycle, response to hormone stimulation, and reproduction sub-networks. The AP on each panel represents the gene contents involved in apoptosis; the CC on each panel refers to the gene content involved in the cell cycle; the HA on each panel is the gene content in response to hormone stimulus; and the RP on each panel represents the gene content involved in reproduction. (TIF) [file pone.0044175.s005.tif]

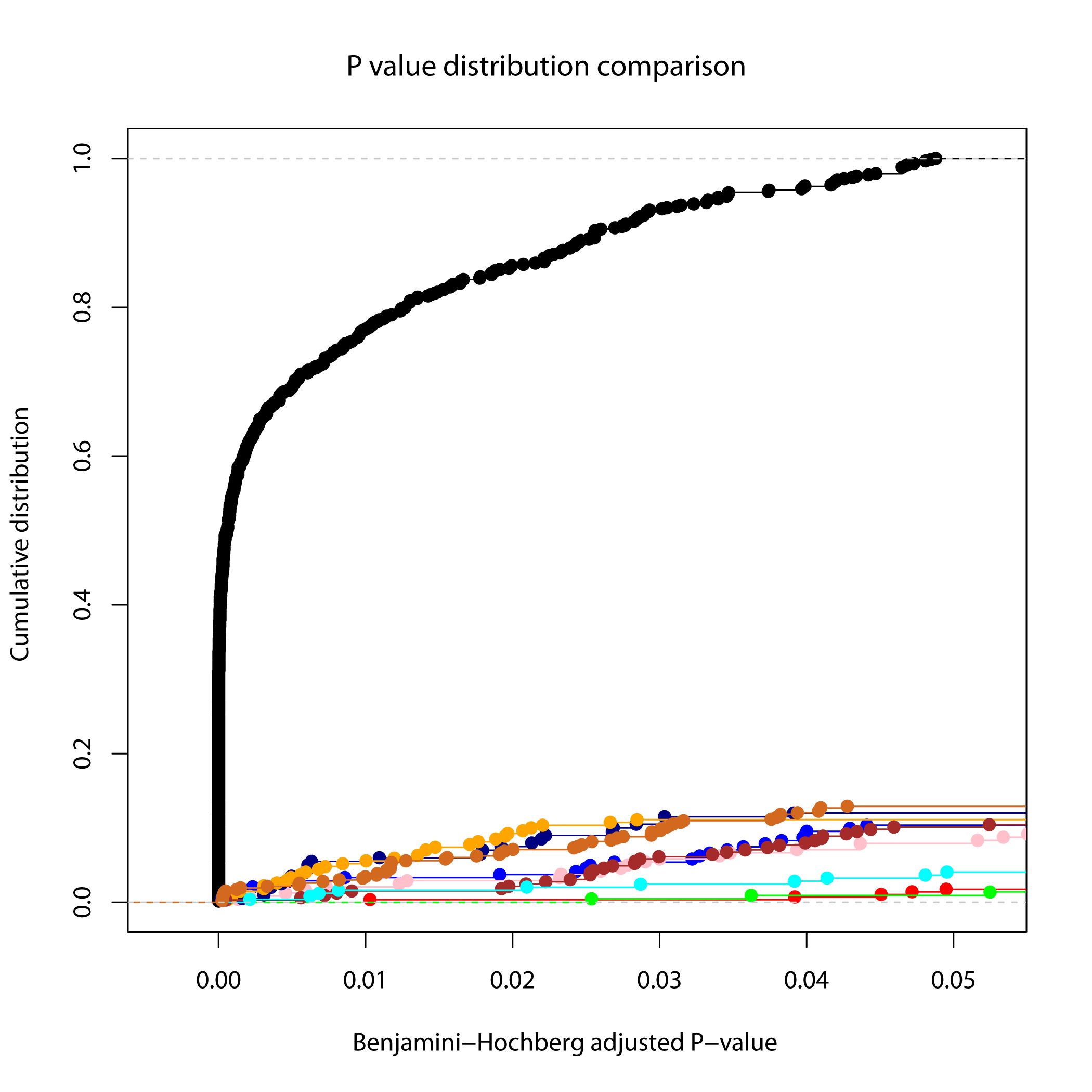

Supplement: Figure S6 — The P- value distribution of functional terms from DAVID for the 112 genes in the ovarian cancer-specific regulatory network and ten gene lists randomly selected from 1257 OVC genes with same number of genes. The empirical cumulative distribution functions (ECDFs) for P-values of different gene datasets. The ECDF curves (black) represent the P-value of the 112 genes in the ovarian cancer-specific regulatory network. The other ten curves represent the P-value of the112 genes randomly selected from 1257 ovarian cancer (OVC)-related genes. For comparison P-values less than 0.05, only the proportions of the P-values less than or equal to 0.05 were plotted. (TIF) [file pone.0044175.s006.tif]
