# Supplementary material for: Distinct and Competitive Regulatory Patterns of Tumor Suppressor Genes and Oncogenes in Ovarian Cancer
Source: PLoS One. 2012 Aug 30;7(8):e44175. doi: 10.1371/journal.pone.0044175 (PMC3431336; doi:10.1371/journal.pone.0044175)
Supplement: Table S2 — The comparison of network topological characteristics in human protein-protein interaction (PPI) among different gene datasets. The results of Kolmogorov-Smirnov test on degree, betweenness centrality and closeness centrality among different gene datasets are included. (DOC) [file pone.0044175.s008.doc]

**Table S2.** Statistical analyses on the topological properties of different gene lists.

| **Gene list (# of genes)** | **Background (# of genes)** | | ***P-* value^a^** |
| --- | --- | --- | --- |
| **Degree** |  | |  |
| Ovarian cancer genes (905) | All human genes (11,654) | | < 2.2 × 10^-16^ |
| Cancer gene census (378) | All human genes (11,654) | | < 2.2 × 10^-16^ |
| TSGs_OCGs_TFs (97) | All human genes (11,654) | | < 2.2 × 10^-16^ |
| TSGs_OCGs_TFs (97) | Ovarian cancer genes (905) | | 7.12 × 10^-8^ |
| TSGs_OCGs_TFs (97) | Cancer gene census (378) | | 3.36 × 10^-2^ |
|  | | |  |
| **Betweenness** **centrality** | | |  |
| Ovarian cancer genes (905) | All human genes (11,654) | | < 2.2 × 10^-16^ |
| Cancer gene census (378) | All human genes (11,654) | | < 2.2 × 10^-16^ |
| TSGs_OCGs_TFs (97) | All human genes (11,654) | | 3.41 × 10^-15^ |
| TSGs_OCGs_TFs (97) | Ovarian cancer genes (905) | | 3.00 × 10^-4^ |
|  | |  | |
| **Closeness centrality** | |  | |
| Ovarian cancer genes (905) | All human genes (11,654) | | < 2.2 × 10^-16^ |
| Cancer gene census (378) | All human genes (11,654) | | < 2.2 × 10^-16^ |
| TSGs_OCGs_TFs (97) | All human genes (11,654) | | 3.41 × 10^-15^ |
| TSGs_OCGs_TFs (97) | Ovarian cancer genes (905) | | 6.99 × 10^-10^ |

The term *All human genes* refers to the 11,654 genes in our integrated human protein-protein interaction (PPI) network. The term *ovarian cancer genes* represents 905 ovarian cancer related genes mapped to human PPI network. Gene list *TSGs_OCGs_TFs* refers to the 97 genes, including 33 tumor suppressor genes (TSGs), 14 oncogenes (OCGs) and 50 transcription factors (TFs) involved in ovarian cancer. The list *Cancer gene census* refers the 378 genes extracted from the Sanger Cancer Gene Census list and mapped to human PPI network [1].

^a^ *P*-values were estimated by Kolmogorov-Smirnov test using R package 2.13.2 [2].

1. Futreal PA, Coin L, Marshall M, Down T, Hubbard T, et al. (2004) A census of human cancer genes. Nat Rev Cancer 4: 177-183.

2. R Development Core Team (2008) R: A Language and Environment for Statistical Computing. R Foundation for Statistical Computing, Vienna, Austria. ISBN 3-900051-07-0, URL http://www.R-project.org.
