# Supplementary material for: Distinct and Competitive Regulatory Patterns of Tumor Suppressor Genes and Oncogenes in Ovarian Cancer
Source: PLoS One. 2012 Aug 30;7(8):e44175. doi: 10.1371/journal.pone.0044175 (PMC3431336; doi:10.1371/journal.pone.0044175)
Supplement: Table S6 — Empirical P- values of network topological characteristics in human protein-protein interaction (PPI) among different gene datasets. The results of randomization on degree, betweenness centrality and closeness centrality among different OVC TSGs, OCGs, and TFs are included. (DOC) [file pone.0044175.s012.doc]

**Table S6.** Empirical P-values of network topological characteristics in human protein-protein interaction of different gene lists.

| **Gene list** | **Degree** | **Betweenness** | **Closeness** |
| --- | --- | --- | --- |
| **TSG** | 0 | 0.0001 | 0 |
| **OCG** | 0.0001 | 0.0005 | 0.0001 |
| **TF** | 0.0013 | 0.08 | 0 |
| **TSG, OCG and TF** | 0 | 0 | 0 |
