# Supplementary material for: Distinct and Competitive Regulatory Patterns of Tumor Suppressor Genes and Oncogenes in Ovarian Cancer
Source: PLoS One. 2012 Aug 30;7(8):e44175. doi: 10.1371/journal.pone.0044175 (PMC3431336; doi:10.1371/journal.pone.0044175)
Supplement: Text S1 — This file includes details of data sources and methods that were used for ovarian cancer (OVC) candidate gene collection and curation. (DOC) [file pone.0044175.s013.doc]

### **Text S1. Detailed Materials and Methods on Ovarian Cancer (OVC) Gene Collection**

Our gene collection on ovarian cancer combined nine data sources including all of the cancer-specific database searches, mutation collection, and manual curation from literature sources. The detail for each data source is described as follows.

COSMIC (Catalogue of Somatic Mutations in Cancer) is currently the most comprehensive resource on somatic mutations in human cancer [1]. We downloaded the COSMIC gene mutation data (version 55, October, 2011) from its public ftp directory. We selected 37,268 cosmic records with tumor source as primary, primary site as ovary and primary histology as carcinoma. For each gene from 37,268 records, we calculated its total sample number (N) and the number of samples with positive mutation records (n). Two strict rules are adapted to isolate high confidential genes. Firstly, the number of samples with positive mutations for the gene is not less than 5; secondly, the percentage between the positive mutation samples and total samples for the gene (n/N) is not less than 3%. As a result, 317 genes satisfied our criteria.

In addition to COSMIC, two other resources (OMIM [2] and GAD [3]) that included the gene mutations on ovarian cancer were also compiled. The OMIM (Online Mendelian Inheritance in Man) database was the first database to collect all known diseases with their genetic component. It provides a precise and comprehensive summary for the clinical and genetic information on ovarian cancer. From its description of ovarian cancer, 19 genes were extracted (Downloaded in October, 2011). The Genetic Association database (GAD) is a resource of summarized human genetic association studies of complex diseases and disorders. It provides 33 genes with positive records for their association with ovarian cancer (Downloaded in October, 2011).

Genes from two cancer gene databases were also included. One is the F-CENSUS [4], an integrated gene resource that includes cancer types, cancer gene types, mutation types and mutation frequencies calculated from the high-throughput mutational screens of cancer genomes. By searching with the keyword “ovarian cancer,” 69 genes were harvested from F-CENSUS server (Downloaded in October, 2011). The other database is Dragon Database for Exploration of Ovarian Cancer Genes (DDOC) [5]. Only 379 genes with experimentally verified information were downloaded from DDOC (Downloaded in October, 2011).

Both small-scale and large-scale experimental results were curated from their origin literatures and supplements. Small-scale experimental data were scattered in the literatures. In our pipeline, we employed strict criterions to collect experimental verified genes on ovarian cancer from Generif literature database (Downloaded in October, 2011) [6]. Genes with two positive generif records related with ovarian cancer were isolated. From this step, only 113 genes were manually curated to be related with ovarian cancer.

There are three genome-wide association (GWA) studies on ovarian cancer. Song et al. [7] first conducted a GWA study on 1,817 cases and 2,353 controls from the UK and identified a new ovarian cancer locus on 9p22.2. The following two GWAs were also conducted in European ancestry cases and controls. Bolton et al. [8] discovered two SNPs at 19p13.11 associated with survival. Goode et al. [9] reported two new loci associated with ovarian cancer including 2q31 and 8q24. In summary, we manually extracted 14 reported susceptibility genes close to the significant SNPs for ovarian cancer from the three papers.

### Recently, The Cancer Genome Atlas Research Network (TCGA) was launched by the National Cancer Institute (NCI) and the National Human Genome Research Institute (NHGRI) to apply genomic technologies to explore the molecular basis of various cancers. One of their main studies on ovarian cancer was recently published in Nature [10]. The released data included somatic mutation, gene expression, DNA copy number variation, gene methylation, and microRNA expression. We compiled six highly confidence gene datasets from its main text and supplementary Tables 5 and 6. Finally, 524 unique genes were summarized from 6 categories of data, including 9 significant somatic mutations, 113 significant focal DNA copy number variations, 168 genetic silenced genes, 193 gene transcription signature related with survival, 22 genes with drug targets and 34 genes in their reported altered pathways. In addition, we also compiled 53 high confidence OVC related genes from a classic review published in Nature Review Cancer [11]. In summary, 1257 candidate genes were integrated, as shown in Table 1 below.

## Table 1 - The ovarian cancer related genes from 14 data sources.

| **Data source** | **# of genes** | **Description** |
| --- | --- | --- |
| DDOC database [5] | 379 | 379 genes with experimentally verified information |
| COSMIC database [1] | 317 | Genes with 5 positive mutations and the proportion of positive mutation samples is over 3% |
| TCGA expression signature genes [10] | 193 | 193 genes to predict overall survival that was defined using the integrated expression data |
| TCGA methylated gene [10] | 168 | 168 epigenetically silenced genes |
| TCGA_26_CNV_regions [10] | 125 | 26 regions of focal amplification that encoded eight or fewer genes |
| Generif database [6] | 113 | Genes with at least 2 positive generif sentences and the gene symbol were exactly matched in the sentences |
| F-census database [4] | 69 | Genes from F-census searched by ovarian cancer |
| Expert review [11] | 51 | Genes collected from the review paper of Nature review cancer on ovarian cancer |
| TCGA pathway analysis [10] | 34 | Genes in significantly altered pathways |
| GAD database [3] | 33 | Genes from GAD with positive association |
| TCGA drug target [10] | 22 | Curated genes with therapeutic compounds |
| OMIM [2] | 19 | Genes summarized from OMIM summary for ovarian cancer |
| GWA studies [7,8,9] | 14 | Significant associated genes from the three GWA studies |
| TCGA significant mutation-related genes [10] | 9 | Significant mutated genes from TCGA Nature paper |

The datasets were sorted by their number of genes in descending order.

### **References**

1. Forbes SA, Bindal N, Bamford S, Cole C, Kok CY, et al. (2011) COSMIC: mining complete cancer genomes in the Catalogue of Somatic Mutations in Cancer. Nucleic Acids Res 39: D945-D950.

2. Amberger J, Bocchini CA, Scott AF, Hamosh A (2009) McKusick's Online Mendelian Inheritance in Man (OMIM). Nucleic Acids Res 37: D793-D796.

3. Becker KG, Barnes KC, Bright TJ, Wang SA (2004) The genetic association database. Nat Genet 36: 431-432.

4. Gong X, Wu R, Zhang Y, Zhao W, Cheng L, et al. (2010) Extracting consistent knowledge from highly inconsistent cancer gene data sources. BMC Bioinformatics 11: 76.

5. Kaur M, Radovanovic A, Essack M, Schaefer U, Maqungo M, et al. (2009) Database for exploration of functional context of genes implicated in ovarian cancer. Nucleic Acids Res 37: D820-823.

6. Aronson AR, Mork JG, Gay CW, Humphrey SM, Rogers WJ (2004) The NLM Indexing Initiative's Medical Text Indexer. Stud Health Technol Inform 107: 268-272.

7. Song H, Ramus SJ, Tyrer J, Bolton KL, Gentry-Maharaj A, et al. (2009) A genome-wide association study identifies a new ovarian cancer susceptibility locus on 9p22.2. Nat Genet 41: 996-1000.

8. Bolton KL, Tyrer J, Song H, Ramus SJ, Notaridou M, et al. (2010) Common variants at 19p13 are associated with susceptibility to ovarian cancer. Nat Genet 42: 880-884.

9. Goode EL, Chenevix-Trench G, Song H, Ramus SJ, Notaridou M, et al. (2010) A genome-wide association study identifies susceptibility loci for ovarian cancer at 2q31 and 8q24. Nat Genet 42: 874-879.

10. The Cancer Genome Atlas Research Network T (2011) Integrated genomic analyses of ovarian carcinoma. Nature 474: 609-615.

11. Bast RC, Jr., Hennessy B, Mills GB (2009) The biology of ovarian cancer: new opportunities for translation. Nat Rev Cancer 9: 415-428.
